# Construct and factorial validity of Neurobehavioral Disorder associated with Prenatal Alcohol Exposure (ND-PAE)

**DOI:** 10.1186/s40359-020-00405-5

**Published:** 2020-05-27

**Authors:** James Ladell Sanders, Nicole Netelenbos, Samuel Ofori Dei

**Affiliations:** Faculty of Health Sciences, University of Lethbridge, 4401 University Dr W, Lethbridge, Alberta T1K 3M4 Canada

**Keywords:** Neurobehavioral Disorder associated with Prenatal Alcohol Exposure (ND-PAE), Fetal Alcohol Spectrum Disorder (FASD), DSM-5, Prenatal alcohol exposure, Diagnosis

## Abstract

**Background:**

ND-PAE, as a condition needing further study, requires validation. Few studies have assessed the validity of ND-PAE with none using a prospective sample.

**Methods:**

Fifty-eight children underwent multidisciplinary FASD assessments and were evaluated for ND-PAE using a prospective, clinical approach. Construct and factorial validity of ND-PAE were assessed, and associations between domains and symptoms described. Post hoc analysis assessed external validity of factors.

**Results:**

ND-PAE demonstrated weak construct validity with variable convergence and divergence within and between symptoms. Factor analysis revealed one strong factor consisting of abilities associated with adaptive behavior and general cognitive ability. Relative contribution of symptoms and domains were variable.

**Conclusion:**

This study provides an evidence-based approach to assessing ND-PAE symptoms and is a starting point to elucidating its neurobehavioral pattern.

## Background

Mental disorders consist of patterns or combinations of abnormal thought, behavior, or affect causing distress or disability [1, 2]. Prenatal alcohol exposure (PAE) can have wide-ranging effects on the developing central nervous system of the fetus. This can result in a host of lifelong neurobehavioral and neurodevelopmental impairments including global intelligence, learning, memory, executive functioning, visual/spatial processing, motor skills, communication, attention, and behavioural and affect regulation [3]. While there is clear evidence of the deleterious effects of PAE, the specific neurobehavioral pattern or combination caused by PAE, should one exist, is not well understood [4].

Early intervention can mitigate these deficits and identification of Fetal Alcohol Spectrum Disorder (FASD) can appropriately inform subsequent health-related treatment [5]. While accurate assessment is of central importance in attaining positive outcomes for clients affected by PAE, there are several limitations to current approaches to FASD assessment. First, FASD assessment is complex given the absence of a specific neurobehavioral profile [4]. The current FASD diagnostic process is painstakingly comprehensive requiring multidisciplinary team meetings and at minimum physicians’ examination and neuropsychological assessment, along with testing from a speech/language pathologist, an occupational therapist, as well as collection of background information and considerable coordination. Although this model for diagnosis is best practice across FASD diagnostic systems [6–10], it can result in limited access in communities with capacity constraints or sustainable funding concerns. In addition, the diagnostic process can be difficult for many patients to navigate, and even more so for those with neurocognitive disabilities to begin with [11]. Second, there are differences between diagnostic systems and health services regarding whether FASD is considered a diagnostic term or a non-diagnostic umbrella term describing a subset of diagnoses including Fetal Alcohol Syndrome, partial Fetal Alcohol Syndrome, and Alcohol-Related Neurodevelopmental Disorder [7, 12]. As a result, patients receiving diagnoses under one system seeking services in another system may be at risk of falling into gaps. Third, with exception of Canadian guidelines [7], current FASD assessment systems do not account for mental health effects of PAE [10, 13].

Neurobehavioral Disorder associated with Prenatal Alcohol Exposure (ND-PAE) is described as a condition requiring further study in the Diagnostic and Statistical Manual for Mental Disorders – Fifth Edition (DSM-5) [14]. Unlike existing FASD diagnostic symptoms, ND-PAE constructs a specific neurobehavioral profile by organizing symptoms into domains and setting symptom criteria to define impairment within those domains. This approach is similar to other mental disorders described in DSM-5. Further, it creates a common diagnostic language across systems and accounts for mental health effects of PAE. Although evidence is not sufficient that it be considered a mental disorder for the purpose of clinical diagnosis [10], ND-PAE does provide opportunity to develop a more efficient diagnostic process and may serve as a platform in the construction of a neurobehavioral profile [13, 15].

### Research assessing the validity of ND-PAE

**Fig. 1:**
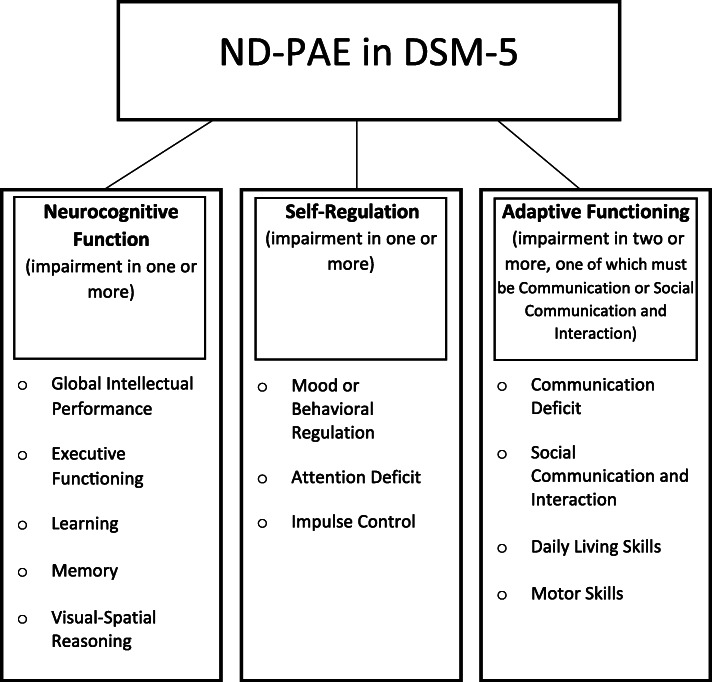
Criteria for ND-PAE based on DSM-5. ND-PAE criteria requires one symptom from each domain of Neurocognitive Function and Self-Regulation and two from Adaptive Function (one of which must be Communication Deficit or Social Communication and Interaction impairment)

Although a number of studies assessing the validity of ND-PAE are underway [10], few published studies have used empirical data from multidisciplinary assessment. The little research on ND-PAE to date suggests that although ND-PAE assesses those areas known to be impacted by PAE, there may be limitations to the organizational structure and criteria of its domains, namely a) Neurocognitive Functioning (one symptom required), b) Self-Regulation (one symptom required), and c) Adaptive Functioning (two symptoms required, one of which must include Communication Deficit or Social Communication and Interaction impairment) (Fig. 1). First, Kable & Coles [16] assessed the internal validity of ND-PAE in a sample of children with Fetal Alcohol Syndrome (FAS) and partial FAS. Given the lack of a clear threshold for symptom severity in ND-PAE, the researchers used criterion levels of 1.5 and 1.0 SD from the mean on norm-referenced measures^1^ and with other descriptive markers for symptom endorsement. Results suggested consistency in ND-PAE symptoms across ages, independent of environmental factors. Reduced threshold of the Adaptive Functioning domain was recommended as it may be too restrictive. Second, Sanders, Hudson Breen, & Netelenbos [17] compared ND-PAE (derived from retrospective chart reviews) to multidisciplinary FASD clinical assessment (using the 2005 Canadian guidelines for diagnosis) [18]. These authors used a threshold of 2.0 SD from the mean on norm-referenced tests and other markers to indicate symptom presence. FASD diagnosis was moderately correlated with ND-PAE. Likewise, these authors concluded that the Adaptive Functioning domain may be overly restrictive. Finally, Johnson et al. [19] identified concordance between ND-PAE criteria and the Alcohol-Related Neurodevelopmental Disorder questionnaire.

Published research thus far has assessed ND-PAE retroactively through examination of existing databases containing norm-referenced test results, clinical notes, and other records. Prospective assessment of ND-PAE, rather, may use a range of procedures including informal testing, direct observations, observer reports, ancillary reports, and clinical interviews [3, 15] that extend beyond norm-referenced testing in identifying symptoms. Further, determination of symptoms and diagnosis is likely associated with, but not dependent upon, norm-referenced test results alone.^2^ DSM-5 criteria in general (including ND-PAE), rather, specifies that symptoms should result in clinically significant distress or impairment in important areas of functioning [14], requiring clinical judgment for interpreting available data. In addition, several ND-PAE domains are most appropriately assessed using norm-referenced testing (i.e., learning, intelligence quotient) while others may be most appropriately assessed through clinical description and judgment (i.e., affect regulation, impulse control), the latter being more difficult to assess retroactively. A prospective clinical assessment of ND-PAE is needed accounting not only for norm-referenced testing, but observations, history, and professional judgment in identifying levels of functional impairment according to ND-PAE criteria.

For the purpose of this study Neurocognitive, Self-Regulation, and Adaptive Functioning are referred to as domains, and its sub-criteria (i.e. Global Intellectual Performance) are described as symptoms.

### Study purpose

The purpose of this study is to use a prospective, integrated clinical approach to ND-PAE assessment to evaluate the construct and factorial validity of ND-PAE and to describe the associations between its domains and symptoms.

## Methods

### Procedure

Children age 7–15 with confirmed PAE underwent multidisciplinary FASD assessments from 2016 to 2019. Clinic team members included clinic/intake coordinator, physician, psychologist, psychometrist, speech-language pathologist, and occupational therapist all trained in FASD assessment. Following intake assessments, medical examination, and specialists’ assessments the entire team convened in determining a diagnosis of FASD and to make recommendations.

Immediately following the FASD diagnostic process team members re-convened to identify ND-PAE symptoms collectively. Team members were reminded that DSM-5 identifies impairment as “clinically significant distress or impairment in social, occupational, or other important areas of functioning”(p.21, 14], therefore, the participant should experience significant distress or impairment in order for a symptom to be endorsed. A participant need not have scored at a certain cutoff (i.e. 2SD) in order to endorse a symptom so long as there was evidence of significant impairment or distress, though norm-referenced scores from the assessment could be used as evidence indicating impairment. The psychologist reviewed each symptom with examples from the DSM-5 (i.e. impairment in executive functioning may consist of “poor planning and organization; inflexibility; difficulty with behavioral inhibition”) [14]. If team members did not agree on symptom endorsement they discussed the evidence leading to the decision until consensus was reached.

Written informed consent was obtained from parents/guardians to use data collected through the clinic. They were informed that participants would not undergo additional testing, that no identifying information would be shared, and that declining or rescinding consent would have no effect on their participation in the FASD clinic nor impact the diagnostic process. This was approved by the University of Lethbridge Office of Research Ethics (2014–055).

### Measures

A standard testing battery (see Appendix) was used along with diagnostic guidelines [7] in the determination of a diagnosis of FASD. The testing battery was derived from recommended measures in the appendix of Cook et al. [7]. For the purpose of this study, additional measures include demographic variables (age, sex), FASD diagnosis, ND-PAE symptoms, and ND-PAE diagnosis.

### Analysis

External validity was examined through cross tabulations and correlation with FASD diagnosis. Construct validity (convergence/divergence) was examined by correlation between domains (Neurocognitive, Self-Regulation, Adaptive Functioning), and by correlation of symptoms within and between domains. Cronbach’s alpha was used to determine the internal consistency of each domain. Factorial validity of ND-PAE domains was assessed with factor analysis (Principal Axis Factoring) with oblique rotation (Promax) given expected correlations between items loading onto different components. Post hoc analysis evaluated the external validity of the components derived from the factor analysis.

## Results

Fifty-eight (58) children (53.4% female) ages 7–15 (mean 10.79(2.3)) participating in the study were assessed between 2016 and 2019. 70.7% (*n* = 41) were diagnosed with FASD and 41.4% (*n* = 24) met criteria for ND-PAE. FASD diagnosis and ND-PAE were strongly correlated (*Phi* = .46) with 67% overall classification accuracy. ND-PAE was the more conservative system with 94.1% specificity (95%CI = 69–100) but 56.1% sensitivity (95%CI = 40–71).

Of the entire sample, 91.4% (*n* = 53) met criteria for impaired neurocognitive functioning (based on at least one of five symptoms) and 87.9% (*n* = 51) for impaired self-regulation (based on one of three symptoms). Impairment in adaptive functioning, however, was identified in much fewer cases (41.4%, *n* = 24) (based on two of four symptoms one of which must be symptoms 1 or 2). Symptom endorsements were most frequently Attention Deficit (84.9%, *n* = 49) and Executive Functioning (77.6%, *n* = 45), and least frequently Daily Living Skills (15.5%, *n* = 9) and Global Intellectual Performance (20.7%, *n* = 12) (Table 1).

**Table 1:** Proportion of clinical sample who met ND-PAE criteria for domains and corresponding symptoms

Neurocognitive Function	(n/%)	Self-Regulation	(n/%)	Adaptive Functioning	(n/%)
Global Intellectual Performance	12/20.7%	Mood or Behavior Regulation	18/31.0%	Communication Deficit	28/48.3%
Executive Functioning	45/77.6%	Attention Deficit	49/84.5%	Social Communication and Interaction	23/39.7%
Learning	38/65.5%	Impulse Control	21/36.2%	Daily Living Skills	9/15.5%
Memory	19/32.8%			Motor Skills	21/36.2%
Visual-Spatial Reasoning	17/29.3%				
**Neurocognitive Impairment**	53/91.4%	**Self-Regulation Impairment**	51/87.9%	**Adaptive Function Impairment**	24/41.4%

***Note:*** Impairment in Neurocognitive Function is manifest in one or more of the corresponding symptoms. Impairment in Self-Regulation is manifest in one or more of the corresponding symptoms. Impairment in Adaptive Function is manifest in two or more of the corresponding symptoms, one of which must be Communication Deficit or Social Communication and Interaction

Table 2 describes *Phi* correlations between all ND-PAE symptoms. Convergent validity of the ND-PAE structure will be demonstrated by stronger correlations between symptoms under the same domain, and divergent validity by weaker correlations between domains and between symptoms across domains.

**Table 2:**
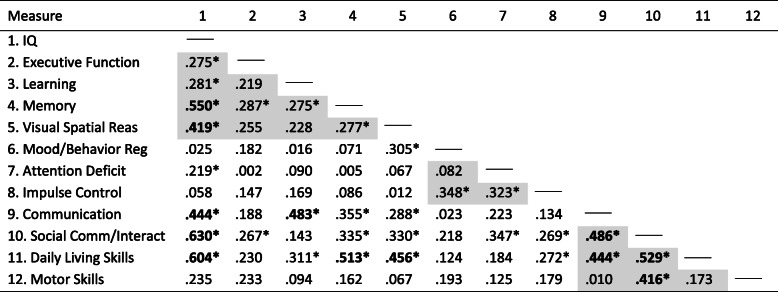
Correlation coefficients (*Phi*) across all ND-PAE symptoms

***Note:*** Shaded grey areas correspond to symptoms that share the same domain. Correlation coefficients above 0.4 are bolded.

*Correlation coefficients are statistically significant at *p* < 0.05

### Convergence

Within the Neurocognitive domain Global Intellectual Performance was significantly correlated (*p < .05*) with all other Neurocognitive symptoms, sharing a very strong association with Memory (*Phi = .55*) and a strong association with Visual-Spatial Reasoning (*Phi = .42*) but weaker associations with Executive Functioning (*Phi = .28*) and Learning (*Phi = .28*). Likewise, Memory shared significant correlations with all other Neurocognitive symptoms (*Phi = .29* for Executive Functioning, *Phi = .28* for Learning and Visual-Spatial Reasoning), albeit weaker than its association with Global Intellectual Performance. There were no significant pairwise correlations between Executive Functioning, Learning, and Visual-Spatial Reasoning (*p > .05*). Cronbach’s alpha was acceptable (.69).

For the Self-Regulation domain, Impulse Control shared significant moderate associations with Mood/Behavioral Regulation (*Phi = .35*) and Attention Deficit (*Phi = .32*). There was no significant correlation between Mood/Behavioral Regulation and Attention Deficit (*p > .05*). Cronbach’s alpha was not acceptable for the Self-Regulation domain (.51), and alpha would increase slightly if the attention symptom was excluded (.52).

Within the Adaptive Functioning domain, Social Communication and Interaction was correlated with all its corresponding symptoms: Communication Deficit (*Phi = .49*), Daily Living Skills (*Phi = .53*), and Motor Skills (*Phi = .42*). Communication Deficit and Daily Living Skills shared a strong correlation (*Phi = .44*) while Motor Skills did not share a significant relation with either (*p > .05*). Cronbach’s alpha for the Adaptive Functioning domain was acceptable (.66), however, would increase if the Motor Skills symptom was removed (.73).

### Divergence

There was no association between the Neurocognitive and Self-Regulation domains (*p > .05*). The Adaptive Functioning domain shared a mild correlation with the Neurocognitive domain (*Phi = .26, p < .05*) and a moderate correlation with Self-Regulation (*Phi = .31, p < .05*).

Symptoms under Neurocognitive Function shared significant associations with 14 of 35 (40%) pairwise comparisons outside this domain. Global Intellectual Performance and Visual-Spatial Reasoning were most frequently associated, each with significant correlations with 4 of 7 (57.1%) non-Neurocognitive symptoms. Self-Regulation symptoms showed greatest divergence, being associated with only 5 of 27 (18.5%) non-Self Regulation symptoms. In contrast, nearly half (15 of 32, 46.9%) of Adaptive Functioning symptoms were associated with non-Adaptive Functioning symptoms. Social Communication and Interaction (6 of 8, 75%) and Daily Living Skills (5 of 8, 62.5%) had the greate st number of pairwise associations with non-Adaptive Functioning Domains. Motor Skills was the only symptom with no pairwise associations across domains but had 1 of 3 (33%) associations within its domain.

### Factorial validity

Principal axis factoring with Promax rotation of the 12 ND-PAE symptoms yielded sufficient sampling adequacy (KMO = .73, Bartlett’s test of sphericity significant (χ2 (66) = 197.03, *p* < .05)) with 64.2% of variance explained. Parallel analysis indicated only Factor 1 met criteria for retention, however, the other factors are explicated in order to assess ND-PAE domains (which are pre-existing). Four factors were extracted consisting of 1) Global Intellectual Performance, Executive Functioning, Memory, Visual-Spatial Reasoning, Social Communication and Interaction, and Daily Living Skills; 2) Learning and Communication Deficit, 3) Attention Deficit, Impulse Control, and Motor Skills, and 4) Mood/Behavioral Regulation (Table 3). Strong associations were derived from symptoms within Factors with the exceptions of Executive Functioning (.383) and Motor Skills (.345). Associations between these sums of symptoms within Factors were then computed demonstrating a strong correlation between Factors 1 and 2, and moderate correlations between Factors 1 and 3, and Factors 3 and 4 (Table 4).

**Table 3:** Principal axis factoring of ND-PAE symptoms

Measure	Factor			
	1	2	3	4
**Neurocognitive Function**				
Global Intellectual Performance	.841			
Executive Functioning	.383			
Learning		.611		
Memory	.615			
Visual-Spatial Reasoning	.517			
**Self-Regulation**				
Mood or Behavioral Regulation				.860
Attention Deficit			.555	
Impulse Control			.529	
**Adaptive Functioning**				
Communication Deficit		.695		
Social Communication & Interaction	.773			
Daily Living Skills	.719			
Motor Skills			.345	

***Note:*** All coefficients in factor loading < .3 are suppressed

**Table 4:** Correlation coefficients (*Spearman rho*) across Factors

Measure	1	2	3	4								
1. Factor 1	—											
2. Factor 2	.463*	—										
3. Factor 3	.334*	.176	—									
4. Factor 4	.213	.023	.317*	—								

*Correlation coefficients that were statistically significant at *p* < .05

### Post hoc analysis

In order to assess the external validity of the components derived from factor analysis, component items were summed and correlated with FASD diagnosis. Strong associations with FASD were derived from components 1 (*ETA* = .51) and 2 (*ETA* = .56) but there were no associations with components 3 (*ETA* = .19) and 4 (*ETA* = .27).

## Discussion

There is no question that PAE engenders the symptoms described in ND-PAE. The construction of these symptoms into a coherent neurobehavioral profile has been problematic, however. This study of ND-PAE criteria appears to be the first to utilize a prospective sample that integrates norm-referenced data with clinical interpretation of impairment and dysfunction in identification of symptoms. Consistent with previous research, results of this study continue to highlight the restrictive nature of the ND-PAE Adaptive Functioning domain. Apart from impairment in Daily Living Skills, impairment in Adaptive Functioning symptoms was relatively common (with Communication Deficit identified fourth most frequently overall and Social Communication and Interaction and Motor Skills tied for fifth). It is the organizational structure of ND-PAE and its domains, however that create restrictions on identification of impairment in Adaptive Functioning. Specifically, this requires endorsement of at least one of Communication Deficit or Social Communication and Interaction Impairment, and endorsement of a second symptom within the construct. Under these criteria, a patient without communication or social interaction impairments would not receive a diagnosis regardless of the neurocognitive, self-regulation, or adaptive functioning impairments that may be present. In essence, these criteria imply communication and social interaction as foundational characteristics of ND-PAE. This is problematic, however, because the PAE neurobehavioral profile is still in question [4] and these two areas of deficit have not been identified as foundational above other areas. Should the Adaptive Functioning domain and its symptoms remain intact, revisions to the number or types of symptoms for domain endorsement are needed.

Results of this study were mixed with regard to convergence of symptoms within domains. There was convergence between some symptoms under their shared domains (i.e. Global Intellectual Performance, Social Communication and Interaction), whereas other symptoms had associations with shared symptoms that were weak or absent (i.e. Executive Functioning, Learning). Internal consistency was acceptable for Neurocognitive and Adaptive Functioning domains but below the acceptable range in the case of Self-Regulation. Individual symptoms of Attention Deficit and Motor Skills adversely affected internal consistency within their respective domains. Likewise, results were mixed in assessing divergence between domains. Overall associations between domains themselves were relatively low suggesting divergence. There was, however, a considerable number of strong associations of symptoms across domains suggesting lack of divergence among symptoms.

There were some symptoms (Executive Functioning and Motor Skills) that were neither associated with symptoms within its shared domain nor other domains. Research has demonstrated clear evidence that PAE can result in Executive Functioning and Motor Skill deficits [20, 21], however, they may not be meaningfully categorized with other symptoms under a larger domain in a diagnostic model such as ND-PAE.

**Fig. 2:**
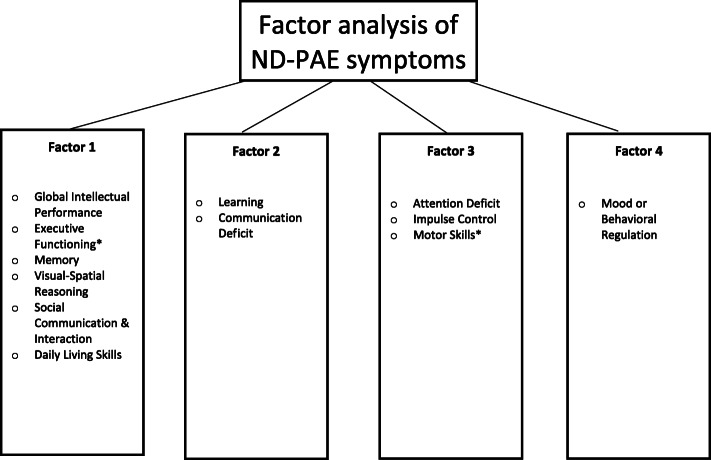
Factor analysis of ND-PAE symptoms. Symptoms marked with * were weakly correlated with their factors (<.4)

An exploratory factor analytic approach was undertaken to assess the factorial validity of ND-PAE. As such, results of the factor analysis should not be overestimated. These results, however, provide a glimpse into which symptoms may cluster similarly to, and differently from, ND-PAE domains, and provide means to assess relative contributions of symptoms within those factors (see Fig. 2).

Factor 1 consisted of symptoms from the Neurocognitive and Adaptive Functioning domains (Global Intellectual Performance, Executive Functioning, Memory, Visual-Spatial Reasoning, Social Communication and Interaction, and Daily Living Skills).

Adaptive behavior refers to skills needed to function effectively and independently for the self, in response to others, and in the larger social environment [22]. The construct of adaptive behavior developed in tandem with the construct of Intellectual Disability and is now integral in its diagnosis. The importance of adaptive behavior in this diagnosis is highlighted in that severity of Intellectual Disability is no longer defined by IQ score, but by degree of adaptive behavior deficits [14]. Definitions of adaptive behavior have developed over time based on assumptions of social competency and adjustment, adaptability to the environment, and managing everyday life demands. Research in the 1970s and 1980s identified common elements between its definitions and subsequent research examined the factor structure of adaptive behavior, consistently identifying four skill areas: conceptual, social, practical, and motor/physical competence. Contemporary assessment of adaptive behavior evaluates the first three domains, with evidence suggesting the motor/physical competence factor may not persist across the lifespan [23].

Various standardized interviews/questionnaires (i.e. VAB3, ABS, ABAS-3, SIB-R) are used to assess adaptive behavior under this general model consisting of conceptual, social, and practical skills. First, conceptual skills refer to competence in areas such as memory, language, problem solving, judgment, and time/money concepts. Second, social skills include interpersonal communication skills, social participation, social problem solving, and social reasoning. Third, the practical domain includes skills associated with health and safety, personal care, following routines, and maintaining household chores [14, 23]. The symptoms comprising Factor 1 appear to generally fit this conceptual (intellect/memory), social (social communication and interaction), and practical (daily living skills) model of adaptive behavior, though this hypothesis requires further validation.

Research has shown an association between PAE and adaptive behavior [24]. Adaptive behavior, adaptive skills, and adaptive functioning, however, has been integrated inconsistently between FASD diagnostic guidelines [7–9]. A standard definition of adaptive behavior is needed in the context of ND-PAE assessment, which should draw on adaptive behavior research already conducted over the past 50 years to lay its foundation rather than beginning anew.

Factor 2 consists of skills acquired through experience and learning. Communication deficits such as receptive or expressive language disorders are relatively common co-morbidities [25], as are learning disorders and deficits in academic achievement, mathematics in particular [15]. Caregiving environments for children with FASD may be variable and/or suboptimal [26], however, and can contribute to delays in development or academic skill acquisition, necessitating differential diagnosis [27]. Delays in academic skills may not manifest until adolescence as course content becomes more complex and classroom expectations increase. While the symptoms of Communication Deficit and Learning are well differentiated from other Factors and symptoms in this study, there is insufficient data to suggest they should be categorized as a stand-alone construct.

Factor 3 consists predominantly of Attention Deficit and Impulse Control, with relatively weak contribution of Motor Skills. Attention Deficit/Hyperactivity Disorder (ADHD) is frequently co-morbid with FASD [28]. These areas are often identified in clinical assessment even in the absence of other deficits. Research has shown neurocognitive differences between ADHD and FASD. For example, children with FASD show problems in underlying cognitive skills and more complex forms of attention (such as shift and encoding), whereas children with ADHD show greater difficulties with sustained and focused attention [29], and the severity of FASD tends to be greater than ADHD [30]. Factor 3 does not speak to external validity of ND-PAE, however, only that set of symptoms are differentiated from other sets of symptoms. Presence of ADHD symptoms may increase risk of false positive identification of ND-PAE and should be interpreted cautiously as central traits in clinical assessment (given that diagnoses of ADHD are relatively commonplace in some countries [31] and may be subject to increased probability of referral).

Factor 4 consists of a sole symptom, Mood and Behavioral Regulation. Many diagnostic systems for FASD do not include mental health assessment. While PAE may be a primary cause of mood and behavioral regulation problems, individuals with PAE at higher risk levels are more likely to experience a range of adversities that can also affect mental health [32].

Among these Factors, only Factors 1 and 2 were associated as clusters with FASD diagnosis. Further, symptoms of Executive Functioning and Motor Skills were weakly associated with their respective factors. While there is evidence from previous research that all ND-PAE symptoms can be adversely impacted by PAE, some areas such as Attention Deficit and Executive Functioning, may have weak differentiating power given that these problems may be commonly present in comorbid issues. Motor Skills shared relatively little association with other symptoms in this study, including those within its shared domain. There is considerable research, however, that PAE is associated with motor skill deficits [21, 33]. Continued assessment of motor skills is needed as it may identify functional impairment or serve as a neurological sign of adverse effects of PAE.

### Recommendations

Future study of ND-PAE is needed and serves two important functions. First, this research can help to elucidate a coherent neurobehavioral profile caused by PAE. Much research to date has identified series’ of deficits associated with PAE [4, 29, 34, 35] but a specific neurobehavioral pattern or combination of symptoms caused by PAE that reliably distinguishes it from other conditions, should one exist, remains elusive. Unlike most mental disorders that are defined by its clusters of symptoms, ND-PAE, FASD, and the like are defined by its cause: PAE. Given the wide ranging effects of PAE on the central nervous system, plus the variable effects of dose, timing, and pattern of PAE, in addition to the compounding effects of other adverse prenatal and postnatal experiences [36], defining a clear neurobehavioral pattern of PAE is formidable. Future research on ND-PAE may clarify patterns of deficit that may lead to identification of central or distinguishing features that may be defined within our current conceptualizations of symptoms (which largely draws from individual standardized assessment) or other theoretical approaches to describing human behavior.

Second, an efficient, descriptive approach to screening and assessment is worth pursuing. FASD diagnosis over the past 20 years has increasingly emphasized the cognitive and behavioral deficits associated with PAE through comprehensive multidisciplinary team assessment [37]. Although this process continues to be important for accurate diagnosis, resource-efficient methods of identification and screening and, eventually, diagnosis are needed. Screening tools for FASD have been developed in the past [19, 38, 39] but this process requires further validation. In addition, a shared language is needed in order to provide equitable supports and treatment and to expedite research. ND-PAE, with its presence in an international taxonomy and its grounding in neurobehavioral evidence, provides opportunity to develop this approach.

Of the factors derived, impairment in Factors 1 and 2 were strongly associated with FASD diagnosis. These Factors should garner particular attention when further developing diagnostic criteria for ND-PAE, elucidating its behavioral patterns, and in constructing its neurobehavioral profile. Additional work is needed in distinguishing the central characteristics of PAE from those that cut across other disorders in order to ensure assessment approaches maintain high specificity. Notwithstanding, all symptoms are grounded in PAE research and could contribute to its diagnostic profile.

### Limitations

The procedure to ND-PAE classification was non-blinded to clients and diagnosis and immediately followed a multidisciplinary assessment meeting where a different set of guidelines were used in determining an FASD diagnosis. Further, the approach is subject to acquiescence bias by clinic team members. There is limited generalizability given that this is a clinical sample from one FASD diagnostic clinic with little variation in team members from the onset of this study. There was low power given the relatively small sample size, therefore, factors derived should be used as a guidepost to future research and not the basis for clinical assessment. This sample does not include non-clinical participants exposed to PAE, however, ND-PAE and its criteria were under evaluation.

## Conclusions

This appears to be the first published study to use a prospective clinical approach to ND-PAE classification. The organization of ND-PAE domains in the DSM-5 demonstrated weak construct and factorial validity. Additional research on the relative contributions of these symptoms is needed. Future research in this area will inform more cost-efficient assessment and screening processes. Further, this research can help elucidate a neurobehavioral pattern, collection of symptoms, or core features of PAE. Finally, we have emphasized the importance of additional PAE research in the context of the ND-PAE domains that extends beyond skills assessed through standardized individual testing and into broader constructs of learning, behavior, and affect, drawing upon existing theories in adaptive behavior, self-regulation, and cognitive psychology.

## Data Availability

The dataset generated during and analysed during the current study are not publicly available due to the sensitive nature of data and small sample size, but relevant variables are available from the corresponding author on reasonable request.

## Appendix

Wechsler Intelligence Scale for Children – Fifth Edition: Canadian [40]

Woodcock Johnson Tests of Achievement – Fourth Edition [41]

California Verbal Learning Test – Children’s Version [42]

Rey Complex Figure Test [43]

Delis-Kaplan Executive Function System [44]

Comprehensive Trail Making Test [45]

Behavior Rating Inventory of Executive Function – Second Edition [46]

Conners 3 [47]

Adaptive Behavior Assessment System – Third Edition [48]

Children’s Depression Inventory – Second Edition [49]

Multidimensional Anxiety Scale for Children – Second Edition [50]

Clinical Evaluation of Language Fundamentals – Fifth Edition [51]

Social Language Development Test [52, 53]

Children’s Communication Checklist – Second Edition [54]

BOT-2 Bruininks-Oseretsky Test of Motor Proficiency – Second Edition [55]

## Abbreviations

ND-PAE: Neurobehavioral Disorder associated with Prenatal Alcohol Exposure
PAE: Prenatal alcohol exposure
FASD: Fetal Alcohol Spectrum Disorder
DSM-5: Diagnostic and Statistical Manual of Mental Disorders – Fifth Edition

## Footnotes

1 A 2 SD threshold is recommended in multidisciplinary diagnostic guidelines [4–8], however, less restrictive thresholds were used to better represent characteristics of the population.

2 DSM-IV-TR defined impairment in general intellectual functioning as usually IQ 70 or below (for diagnosis of Mental Retardation) with accompanying adaptive functioning deficits. For a DSM-IV-TR diagnosis of Learning Disorder, academic achievement is usually 2 standard deviations below IQ. These specific references to norm-based thresholds of impairment or discrepancies were removed in DSM-5 in lieu of more functional criteria.
